# The correlation between fruit intake and all-cause mortality in hypertensive patients: a 10-year follow-up study

**DOI:** 10.3389/fnut.2024.1363574

**Published:** 2024-03-22

**Authors:** Chuang Sun, Jie Li, Zeyuan Zhao, Shupeng Ren, Yue Guan, Miaoan Zhang, Tianfeng Li, Linglin Tan, Qiying Yao, Liang Chen

**Affiliations:** ^1^Department of Cardiology, The Second Affiliated Hospital of Dalian Medical University, Dalian, China; ^2^General Practice Department, The First Affiliated Hospital of Yangtze University, Jingzhou, China; ^3^Department of Physiology, Dalian Medical University, Dalian, China

**Keywords:** fruit consumption, apple consumption, banana consumption, hypertension, all-cause mortality

## Abstract

**Objective:**

Extensive research has consistently shown the beneficial impact of fruit consumption on overall health. While some studies have proposed a potential association between fruit consumption and hypertension management, the influence of fruit consumption on mortality rates among hypertensive individuals remains uncertain. Consequently, aim of this study is to evaluate whether fruit consumption is associated with all-cause mortality among hypertensive patients.

**Methods:**

Data were obtained from the National Health and Nutrition Examination Survey (NHANES), conducted between 2003 and 2006. Ten-year follow-up data from the National Death Index (NDI) were used to assess all-cause mortality. Cox proportional hazard model was utilized to explore the impact of fruit intake on all-cause mortality among hypertensive individuals.

**Results:**

The study included a cohort of 2,480 patients diagnosed with hypertension, and during the follow-up period, a total of 658 deaths from various causes were recorded. The COX regression analysis demonstrated that hypertensive patients who consumed apples three to six times per week exhibited a significantly reduced risk of all-cause mortality (HR = 0.60, 95%CI: 0.45–0.78, *p* < 0.001) in comparison to those who consumed apples less than once per month. Likewise, consuming bananas three to six times per week also led to a comparable outcome (HR = 0.76, 95%CI: 0.59–0.97, *p* = 0.027). Moreover, Combined consumption of bananas and apples three to six times per week exhibited a noteworthy decrease in all-cause mortality (HR = 0.57, 95%CI: 0.39–0.84, *p* = 0.005) when compared to individuals who consumed these fruits less frequently. Conversely, no significant association was found between the consumption of other fruits, including pears, pineapples, and grapes, and all-cause mortality.

**Conclusion:**

The study discovered that moderate consumption of apples and bananas was associated with a reduced risk of all-cause mortality in patients with hypertension.

## Introduction

Fruits play a pivotal role in promoting a nourishing diet on a global scale (1). The Dietary Guidelines for Americans 2015–2020 advocate for the inclusion of fruits in dietary patterns, with a particular emphasis on the consumption of whole fruits (2). Extensive research has consistently demonstrated a significant inverse association between the intake of substantial quantities of fruit and the risk of developing cardiovascular disease, obesity, diabetes, cancer, and stroke (3–7). Moreover, studies have indicated that fruit consumption can effectively mitigate the risk of all-cause mortality in individuals with diabetes and specific types of cancer (8, 9).

Hypertension is a widely prevalent cardiovascular disorder. The World Health Organization’s global report on Hypertension reveals that approximately one third of the global population experiences this condition, resulting in over 10 million deaths annually attributed to elevated systolic blood pressure, with a substantial annual mortality rate (10). Hypertension remains the most significant modifiable risk factor for global all-cause mortality (11). The reportprimarily emphasizes the mitigation of hypertension through lifestyle modifications, with particular attention given to the impact of dietary patterns on blood pressure (10). Both the Dietary Approaches to Stop Hypertension and Mediterranean diets are recognized in the scientific community as effective dietary patterns for reducing high blood pressure (12–16). Both diets emphasize the consumption of fruits in their recommended meal plan (12–16).

Fruit-based diets, particularly those including apples, bananas, pears, and grapes, can have a positive impact on human health due to their high nutrient content. These fruits are rich in antioxidants, vitamins, and potassium (17). These nutrients can reduce blood pressure by improving endothelial function, modulating stress reflex sensitivity, and increasing antioxidant activity (18). Additionally, antioxidants can hinder the generation of reactive oxygen species, these species can scavenge excess and harmful free radicals that are produced during normal metabolism, prevent DNA damage, and ultimately reduce all-cause mortality (19). Nevertheless, a consensus regarding the optimal quantity of fruit intake has yet to be reached. Discrepancies in nutritional guidelines for fruit consumption persist across different nations. The World Health Organization advocates for the consumption of five fruit servings, the U.S. Dietary Guideline suggests at least 2 servings, whereas the Chinese Dietary Guideline suggests consuming 2.5–4.5 servings (20–22). Additionally, limited research has been conducted on the association between fruits intake and mortality rates among individuals with hypertension. Thus, the objective of this study is to assess the relationship between fruit consumption and all-cause mortality within a cohort of hypertensive patients.

## Methods

### Study design and population

**Figure 1:**
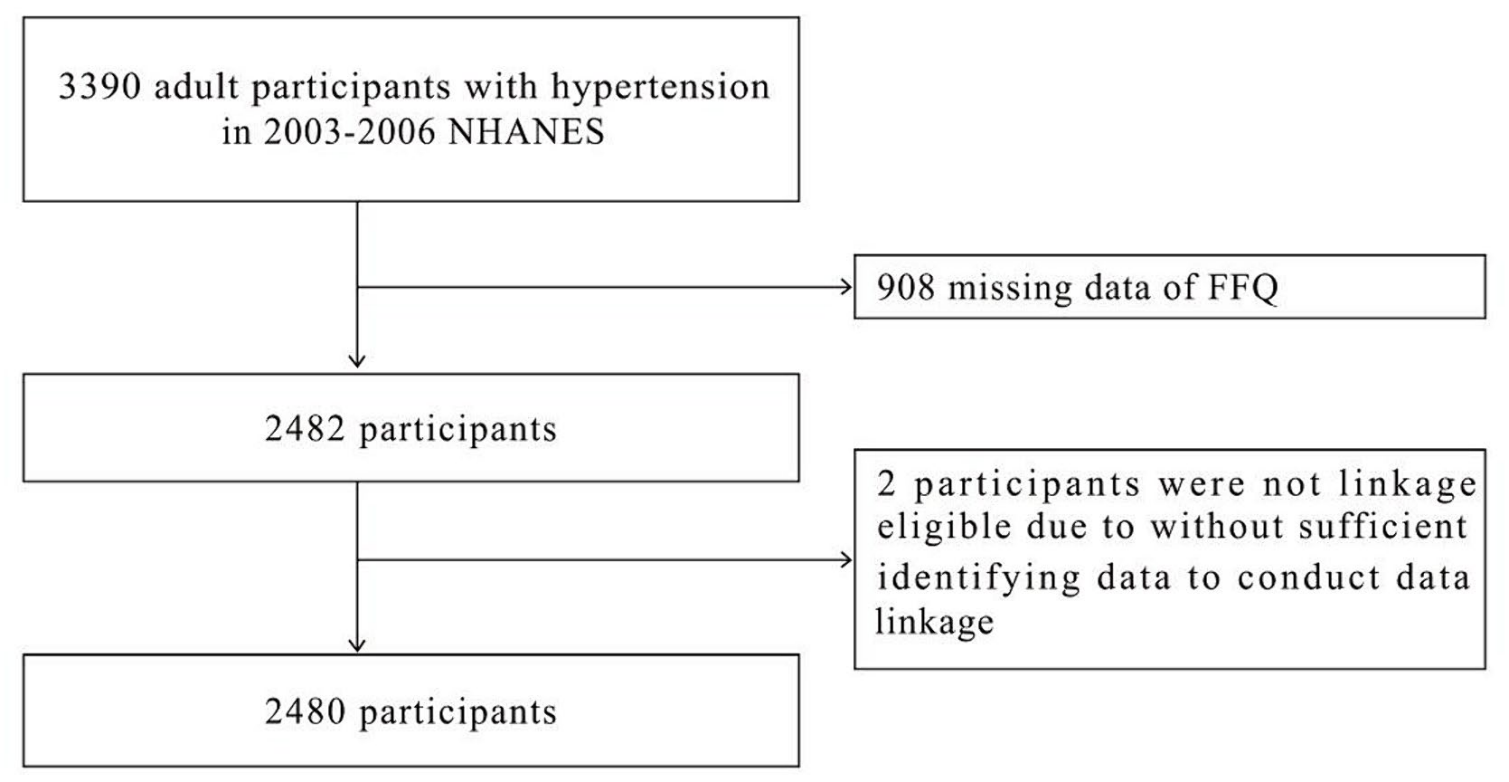
Flowchart of participant selection.

Source of study population was from the National Health and Nutrition Examination Survey (NHANES), a public database that monitors human health conditions and nutrition across ethnic groups in the United States through continuous updates. NHANES obtained resident samples of all races in the United States through multi-stage stratified sampling, including people of all ages from all regions of the country. Therefore, NHANES data is a representative reflection of the health status of United States residents. Researchers can obtain NHANES data for free from the official website (Supplementary Table S1). The Ethics Review Board of the National Center for Health Statistics had approved the NHANES study. All participants provided written informed consent, allowing researchers to use the data as long as the data source is identified in the findings. The NHANES database included 11,183 adult participants from diverse ethnic and geographic backgrounds, between 2003 and 2006. Hypertension was identified through a computer-assisted personal interview system and a questionnaire. 3,390 participants were identified as having hypertension, while 908 participants were excluded due to not completing the Food Frequency Questionnaire (FFQ). Two participants lacked sufficient information to link to the National Death Index (NDI) data, resulting in missing follow-up survival status results and their exclusion from the study. Ultimately, 2,480 participants were enrolled in the study, as illustrated in Figure 1.

### Fruit consumption assessment

The Food Frequency Questionnaire (FFQ) is a tool used to collect information about food intake frequency in the past 12 months, including fruit consumption habits. Participants in this study were asked about their average intake of apples over the last year by responding to the prompt: “How often did you eat apples/ bananas/ pears/pineapples/grapes?” Their eating habits were then analyzed. Fruit consumption data was obtained via an FFQ, which categorized intake levels as follows: unknow, less than once /month, 1–3 times/month, 1–2 times/ week, three to six times/ week, or once or more /day for apples, pears, bananas, pineapples, and grapes.

### Outcome assessment

All-cause mortality was examined using data from the National Death Index (NDI), which recorded death information for study participants from the survey’s commencement through December 31, 2019. The NDI, published by the National Center for Health Statistics, a United States government agency, connects NHANES data with mortality records. Data perturbation techniques were utilized in the NDI to reduce the risk of participant re-identification and maintain patient confidentiality. Study period concludes 10 years after enrollment. For deceased patients, the follow-up duration since their interview was computed based on the quarter and year of their passing. For living patients, follow-up duration was determined using the end of the follow-up period. All NDI data used in this study were obtained from publicly released documents on the website (Supplementary Table S1).

### Covariates assessment

The collection of baseline patient data was facilitated by a computer-assisted personal interview system and a questionnaire survey. This comprehensive dataset encompassed various demographic factors such as gender, age, race, education level, ratio of family income to poverty, as well as health-related information including smoking and alcohol consumption, presence of hypercholesterolemia, diabetes, cardiovascular disease, stroke, lung disease, liver disease, and failing kidneys. Ethnicity was determined by categorizing patients as Mexican Americans, non-Hispanic Blacks, non-Hispanic whites, other Hispanics, or others. A smoking history of at least 100 cigarettes smoked throughout one’s lifetime was used as the criterion for identifying individuals as smokers. A drinker was operationally defined as an individual who had consumed a minimum of 12 cups alcoholic beverages within the preceding 12-month period. The presence of hypertension, hypercholesterolemia, diabetes, stroke, liver disease, and renal failure was ascertained based on prior diagnoses made by the participant’s healthcare provider. Participants who responded affirmatively to any of the following inquiries were categorized as having cardiovascular disease: “Have you ever been diagnosed with congestive heart failure, coronary heart disease, angina pectoris, or a heart attack by a healthcare professional?”

### Statistical analysis

The median (first quartile, third quartile) was employed for continuous variables that did not adhere to a normal distribution. Categorical variables were expressed as numbers (percentages). The comparison of non-normally distributed continuous variables between two groups was conducted using the rank sum test. Furthermore, Chi-square tests were utilized to compare categorical variables between two groups. Cox proportional hazard model was utilized to examine the impact of fruit intake on all-cause mortality. Model 1 did not adjust any covariates. Model 2 made adjustments for gender, age, race, education level, and ratio of family income to poverty rate. In Model 3, covariates were selected based on the principle of covariate screening: confounding factors were compared before and after adding them to the regression model, and those resulting in a change in *p* value greater than 10% were included. Adjusting factors in the final Model 3 included sex, age, race, education level, ratio of family income to poverty rate, smoking history, history of hypercholesterolemia, diabetes, history of cardiovascular disease, stroke, lung disease, and history of failing kidneys. Before COX regression, collinearity screening was carried out for all covariates. The results indicated that the Variance Inflation Factor (VIF) for all covariates was less than 5. Splines smoothing plots were utilized to examine the linear or nonlinear correlation between the frequency of consuming different kinds of fruits and all-cause mortality. Additionally, stratified analyses were performed to confirm the consistency of the outcomes in the presence of different diseases. In addition, further elucidating the relationship between fruit intake and mortality was done by using a three-dimensional histogram and a time-mortality curve. The analysis of data was conducted using the R language package and EmPower software.^1^ *p*-values less than 0.05 are considered statistically significant.

## Results

### Baseline characteristics of the participants

A total of 2,480 participants met the study’s exclusion criteria and were included (Figure 1). The baseline characteristics of each participant are presented in Table 1. During the follow-up period, there were 1822 individuals who survived and 658 who died. The survivor group consisted of 44.5% males and 55.5% females. 15.8% of the population identified as Mexican American, 1.9% as other Hispanic, 53.4% as non-Hispanic white, 25% as non-Hispanic black, and 3.8% as belonging to other race. In regards to educational attainment, 45.4% of the population had education beyond high school. Concerning income, 40.3% of households had an income to poverty ratio greater than 3. Of the participants in survival group, 62.1% reported consuming alcohol, 48.9% smoked cigarettes, 47.3% had hypercholesterolemia, 17.8% suffered from diabetes, 14.2% from cardiovascular disease, 5.3% from stroke, 18.6% from lung disease, 4.1% from liver disease, and 3.3% from failing kidneys. In the group that experienced death, 55.5% were male and 44.5% were female. By race, 12.3% identified as Mexican American, 1.1% as other Hispanic, 65.5% as non-Hispanic white, 19.3% as non-Hispanic black, and 1.8% as other race. In terms of educational attainment, 34.2% possessed a high school education or higher. Regarding income, 24.8% had a ratio of family income to poverty exceeding 3. Of all participants in all-cause death group, 59.7% reported alcohol consumption, 62.8% reported smoking, 52.4% had hypercholesterolemia, 28.4% had diabetes, 37.8% had cardiovascular disease, 18.1% had a history of stroke, 26.1% suffered from lung disease, 4% experienced liver disease, and 10.9% had failing kidneys.

**Table 1:** Baseline characteristics of participants.

	All	Survival	All-cause death	*p*
Number	2,480	1822	658	
**Age, years**	64.0 (51.0–74.0)	60.0 (46.0–69.0)	75.0 (66.0–80.0)	<0.001
**Sex**				<0.001
Male	1,176 (47.4)	811 (44.5)	365 (55.5)	
Female	1,304 (52.6)	1,011 (55.5)	293 (44.5)	
**Race**				<0.001
Mexican American	369 (14.9)	288 (15.8)	81 (12.3)	
Other Hispanic	42 (1.7)	35 (1.9)	7 (1.1)	
Non-Hispanic White	1,404 (56.6)	973 (53.4)	431 (65.5)	
Non-Hispanic Black	583 (23.5)	456 (25.0)	127 (19.3)	
Other race	82 (3.3)	70 (3.8)	12 (1.8)	
**Education level**				<0.001
<High school	747 (30.1)	494 (27.1)	253 (38.4)	
High school graduate or general equivalency diploma	680 (27.4)	501 (27.5)	179 (27.2)	
>High school	1,052 (42.4)	827 (45.4)	225 (34.2)	
Unknown	1(0.1)	0 (0.0)	1 (0.2)	
**Ratio of family income to poverty**				<0.001
≤1	372 (15.0)	266 (14.6)	106 (16.1)	
1–3	1,094 (44.1)	745 (40.9)	349 (53.0)	
>3	898 (36.2)	735 (40.3)	163 (24.8)	
Unknown	116 (4.7)	76 (4.2)	40 (6.1)	
**Alcohol use**				0.278
No	955 (38.5)	690 (37.9)	265 (40.3)	
Yes	1,525 (61.5)	1,132 (62.1)	393 (59.7)	
**Smoking**				<0.001
No	1,176 (47.4)	931 (51.1)	245 (37.2)	
Yes	1,304 (52.6)	891 (48.9)	413 (62.8)	
**Hypercholesterolemia**				0.024
No	1,273 (51.3)	960 (52.7)	313 (47.6)	
Yes	1,207 (48.7)	862 (47.3)	345 (52.4)	
**Diabetes**				<0.001
No	1968 (79.4)	1,497 (82.2)	471 (71.6)	
Yes	512 (20.6)	325 (17.8)	187 (28.4)	
**Cardiovascular Disease**				<0.001
No	1972 (79.5)	1,563 (85.8)	409 (62.2)	
Yes	508 (20.5)	259 (14.2)	249 (37.8)	
**Stroke**				<0.001
No	2,264 (91.3)	1725 (94.7)	539 (81.9)	
Yes	216 (8.7)	97 (5.3)	119 (18.1)	
**Lung Disease**				<0.001
No	1969 (79.4)	1,483 (81.4)	486 (73.9)	
Yes	511 (20.6)	339 (18.6)	172 (26.1)	
**Liver Condition**				0.902
No	2,380 (96.0)	1748 (95.9)	632 (96.0)	
Yes	100 (4.0)	74 (4.1)	26 (4.0)	
**Failing Kidneys**				<0.001
No	2,348 (94.7)	1762 (96.7)	586 (89.1)	
Yes	132 (5.3)	60 (3.3)	72 (10.9)	
**Apple intake**				<0.001
<1 times/month	953 (38.4)	670 (36.8)	283 (43.0)	
1–3 times/month	603 (24.3)	466 (25.6)	137 (20.8)	
1–2 times/week	423 (17.1)	317 (17.4)	106 (16.1)	
3–6 times/week	341 (13.8)	268 (14.7)	73 (11.1)	
≥1 times/day	101 (4.1)	66 (3.6)	35 (5.3)	
Unknow	59 (2.4)	35 (1.9)	24 (3.6)	
**Banana intake**				<0.001
<1 times/month	532 (21.5)	401 (22.0)	131 (19.9)	
1–3 times/month	500 (20.2)	392 (21.5)	108 (16.4)	
1–2 times/week	508 (20.5)	385 (21.1)	123 (18.7)	
3–6 times/week	592 (23.9)	429 (23.5)	163 (24.8)	
≥1 times/day	296 (11.9)	183 (10.0)	113 (17.2)	
Unknow	52 (2.1)	32 (1.8)	20 (3.0)	
**Pear intake**				0.689
<1 times/month	1,571 (63.3)	1,165 (63.9)	406 (61.7)	
1–3 times/month	498 (20.1)	367 (20.1)	131 (19.9)	
1–2 times/week	248 (10.0)	177 (9.7)	71 (10.8)	
3–6 times/week	85 (3.4)	61 (3.3)	24 (3.6)	
≥1 times/day	23 (0.9)	16 (0.9)	7 (1.1)	
Unknow	55 (2.2)	36 (2.0)	19 (2.9)	
**Pineapple intake**				0.761
<1 times/month	1722 (69.4)	1,260 (69.2)	462 (70.2)	
1–3 times/month	500 (20.2)	378 (20.7)	122 (18.5)	
1–2 times/week	151 (6.1)	110 (6.0)	41 (6.2)	
3–6 times/week	43 (1.7)	30 (1.6)	13 (2.0)	
≥1 times/day	12 (0.5)	9 (0.5)	3 (0.5)	
Unknow	52 (2.1)	35 (1.9)	17 (2.6)	
**Grape intake**				0.073
<1 times/month	1,074 (43.3)	758 (41.6)	316 (48.0)	
1–3 times/month	766 (30.9)	580 (31.8)	186 (28.3)	
1–2 times/week	359 (14.5)	273 (15.0)	86 (13.1)	
3–6 times/week	170 (6.9)	131 (7.2)	39 (5.9)	
≥1 times/day	44 (1.8)	34 (1.9)	10 (1.5)	
Unknow	67 (2.7)	46 (2.5)	21 (3.2)	

Continuous variables are presented as the median (Q1, Q3), and categorical variables are summarized as numbers (%). Differences in baseline characteristics were compared with the chi-square test for categorical variables and rank sum test for continuous variables. Q1, first quartile; Q3, third quartile.

### Relationship between fruit consumption and all-cause mortality

**Figure 2:**
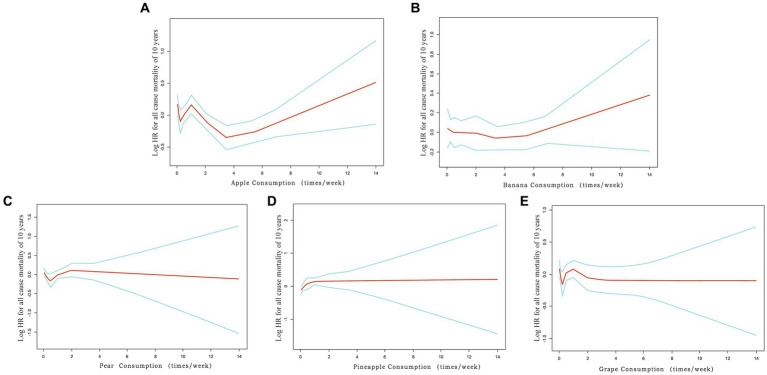
Splines smoothing plots of relationship between fruit consumption and all-cause mortality. **(A)** Splines smoothing plots of relationship between apple intake and all-cause mortality in patients with hypertension. **(B)** Banana intake. **(C)** Pear intake. **(D)** Pineapple intake. **(E)** Grape intake. HR has been fully adjusted for gender, age, race, education level, ratio of family income to poverty rate, smoking, hypercholesterolemia, diabetes, cardiovascular disease, stroke, lung disease, failing kidneys. HR, hazard ratio.

The study examined the association between fruit consumption and all-cause mortality. The findings from splines smoothing plots analysis, as depicted in Figure 2, illustrated a decreasing trend in the risk of all-cause mortality when consuming apples and bananas three to six times per week. However, no significant changes in the risk of all-cause mortality were observed for other fruits, regardless of the intake levels.

The findings from the multivariable adjusted Model 3 reveal that individuals who consume apples or bananas three to six times per week have a reduced risk of all-cause mortality by 40% (HR = 0.60, 95% CI: 0.45–0.78, *p* < 0.001) and 24% (HR = 0.76, 95% CI: 0.59–0.97, *p* = 0.027), respectively, compared to those who consume them less than once per month (Table 2). However, no statistically significant correlation was found between other frequency of consumption of apples and bananas and the risk of all-cause mortality. Furthermore, it is worth noting that no significant association is identified between the consumption of other fruits (including pears, pineapples, and grapes) and all-cause mortality.

**Table 2:** Hazard ratios and confidence intervals of all-cause mortality for fruit consumption.

		Model 1	Model 2	Model 3
	Number	HR (95%CI) *p*	HR (95%CI) *p*	HR (95%CI) *p*
**Apple intake**				
<1 times/month	953	Ref	Ref	Ref
1–3 times/month	603	0.73 (0.60, 0.90) 0.003**	0.79 (0.64, 0.98) 0.032*	0.81 (0.66, 1.01) 0.056
1–2 times/week	423	0.82 (0.66, 1.03) 0.082	0.80 (0.63, 1.00) 0.055	0.86 (0.68, 1.08) 0.199
3–6 times/week	341	0.69 (0.53, 0.89) 0.004**	0.56 (0.43, 0.73) <0.001***	0.60 (0.45, 0.78) <0.001***
≥1 times/day	101	1.19 (0.84, 1.69) 0.326	0.91 (0.63, 1.30) 0.592	0.94 (0.65, 1.35) 0.724
**Banana intake**				
<1 times/month	532	Ref	Ref	Ref
1–3 times/month	500	0.86 (0.67, 1.11) 0.240	0.78 (0.60, 1.01) 0.062	0.80 (0.61, 1.05) 0.105
1–2 times/week	508	0.98 (0.77, 1.26) 0.896	0.79 (0.61, 1.03) 0.077	0.82 (0.63, 1.06) 0.124
3–6 times/week	592	1.14 (0.90, 1.43) 0.274	0.74 (0.58, 0.94) 0.013*	0.76 (0.59, 0.97) 0.027*
≥1 times/day	296	1.69 (1.31, 2.17) <0.001***	0.95 (0.73, 1.23) 0.675	1.01 (0.77, 1.31) 0.958
**Pear intake**				
<1 times/month	1,571	Ref	Ref	Ref
1–3 times/month	498	1.04 (0.85, 1.26) 0.718	0.83 (0.68, 1.02) 0.080	0.82 (0.67, 1.01) 0.058
1–2 times/week	248	1.13 (0.88, 1.45) 0.341	0.93 (0.71, 1.21) 0.583	0.92 (0.70, 1.20) 0.538
3–6 times/week	85	1.14 (0.76, 1.73) 0.524	0.92 (0.59, 1.41) 0.690	0.87 (0.56, 1.34) 0.527
≥1 times/day	23	1.17 (0.56, 2.48) 0.674	1.21 (0.57, 2.58) 0.613	1.27 (0.60, 2.72) 0.535
**Pineapple intake**				
<1 times/month	1722	Ref	Ref	Ref
1–3 times/month	500	0.90 (0.74, 1.10) 0.319	0.96 (0.78, 1.18) 0.684	1.00 (0.82, 1.24) 0.963
1–2 times/week	151	1.05 (0.76, 1.44) 0.774	1.16 (0.83, 1.61) 0.391	1.00 (0.71, 1.40) 0.996
3–6 times/week	43	1.18 (0.68, 2.05) 0.553	1.33 (0.76, 2.31) 0.314	1.48 (0.84, 2.58) 0.172
≥1 times/day	12	0.87 (0.28, 2.71) 0.810	0.78 (0.25, 2.46) 0.675	0.86 (0.27, 2.78) 0.802
**Grape intake**				
<1 times/month	1,074	Ref	Ref	Ref
1–3 times/month	766	0.81 (0.68, 0.97) 0.023*	0.84 (0.70, 1.02) 0.072	0.84 (0.70, 1.02) 0.076
1–2 times/week	359	0.79 (0.62, 1.00) 0.047*	1.00 (0.78, 1.28) 0.978	1.01 (0.79, 1.30) 0.927
3–6 times/week	170	0.76 (0.55, 1.06) 0.111	0.76 (0.54, 1.07) 0.116	0.75 (0.54, 1.06) 0.109
≥1 times/day	44	0.74 (0.40, 1.40) 0.358	0.82 (0.42, 1.60) 0.566	0.80 (0.41, 1.56) 0.511

Model 1: Not adjusted.

Model 2: Adjusted for gender, age, race, education level, ratio of family income to poverty rate.

Model 3: Adjusted for gender, age, race, education level, ratio of family income to poverty rate, smoking, hypercholesterolemia, diabetes, cardiovascular disease, stroke, lung disease, failing kidneys.

CI, confidence interval; HR, hazard ratio; Ref, reference. *, *p* < 0.05, **, *p* < 0.01, ***, *p* < 0.001.

### Relationship between apple intake and all-cause mortality

**Figure 3:**
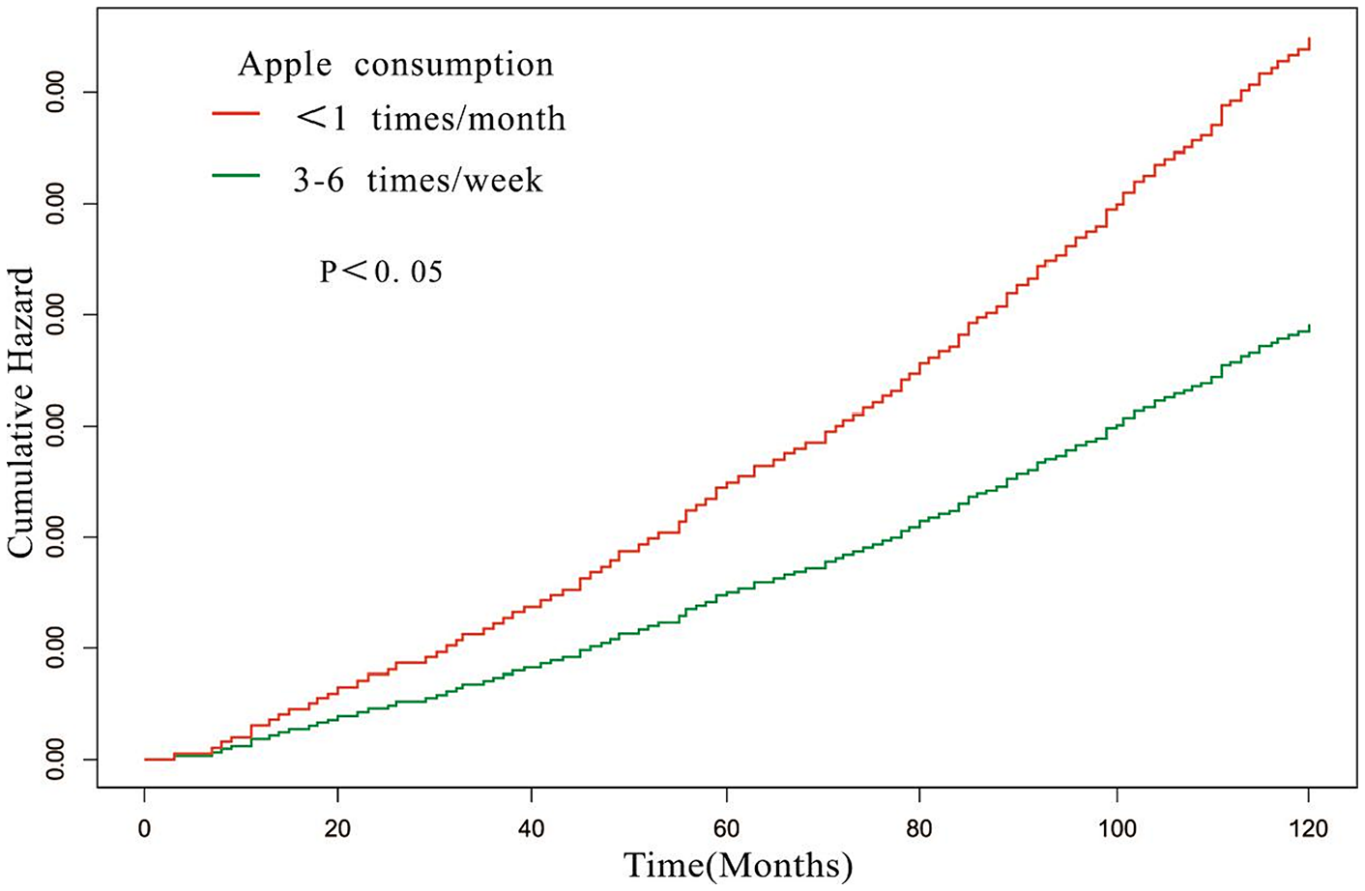
Time-mortality curve of the relationship between apple consumption and all-cause mortality. Adjusted for gender, age, race, education level, ratio of family income to poverty rate, smoking, hypercholesterolemia, diabetes, cardiovascular disease, stroke, lung disease, failing kidneys.

Subgroup analyses revealed that the association between apple consumption and all-cause mortality among individuals with hypertension remained unaffected by the presence or absence of comorbidities (all *p* values for the interaction were > 0.05) (refer to Table 3). Over the course of a 10-year follow-up period, our findings demonstrated that hypertensive individuals who consumed apples three to six times per week had a significantly lower risk of mortality compared to those who consumed them less than once a month. This observation is substantiated by the risk ratio results for all-cause mortality derived from the fully adjusted model, as illustrated in Figure 3 (log-rank test *p* < 0.05).

**Table 3:** Stratified analysis of relationship between apple intake and all-cause mortality.

		HR (95%CI)					P inter
	Number	<1 times/month	1–3 times/month	1–2 times/week	3–6 times/week	≥1 times/day	
**Hypercholesterolemia**							0.843
No	1,273	Ref	0.75 (0.55, 1.03)	0.93 (0.66, 1.31)	0.60 (0.41, 0.89)	0.91 (0.50, 1.68)	
Yes	1,207	Ref	0.84 (0.62, 1.12)	0.77 (0.56, 1.07)	0.56 (0.38, 0.83)	0.95 (0.60, 1.52)	
**Diabetes**							0.913
No	1968	Ref	0.76 (0.59, 0.97)	0.83 (0.63, 1.09)	0.58 (0.42, 0.81)	0.99 (0.63, 1.58)	
Yes	512	Ref	0.94 (0.62, 1.43)	0.95 (0.59, 1.51)	0.59 (0.36, 0.95)	0.96 (0.52, 1.79)	
**Cardiovascular Disease**							0.797
No	1972	Ref	0.78 (0.59, 1.02)	0.80 (0.60, 1.08)	0.58 (0.42, 0.82)	1.06 (0.67, 1.67)	
Yes	508	Ref	0.84 (0.59, 1.19)	1.00 (0.68, 1.45)	0.56 (0.36, 0.89)	0.81 (0.43, 1.53)	
**Stroke**							0.242
No	2,264	Ref	0.78 (0.62, 0.99)	0.78 (0.60, 1.01)	0.60 (0.45, 0.80)	1.12 (0.75, 1.67)	
Yes	216	Ref	0.88 (0.53, 1.46)	1.35 (0.78, 2.34)	0.46 (0.20, 1.05)	0.73 (0.27, 1.96)	
**Lung Disease**							0.180
No	1969	Ref	0.79 (0.62, 1.01)	0.87 (0.67, 1.14)	0.56 (0.41, 0.77)	0.83 (0.55, 1.25)	
Yes	511	Ref	0.86 (0.56, 1.33)	0.84 (0.53, 1.34)	0.71 (0.40, 1.27)	3.46 (1.38, 8.67)	
**Liver Condition**							0.465
No	2,380	Ref	0.82 (0.66, 1.01)	0.87 (0.68, 1.10)	0.61 (0.46, 0.80)	0.93 (0.63, 1.36)	
Yes	100	Ref	2.01 (0.42, 9.56)	1.10 (0.22, 5.57)	0.62 (0.06, 6.45)	4.79 (0.45, 50.84)	
**Failing Kidneys**							0.920
No	2,348	Ref	0.81 (0.65, 1.02)	0.86 (0.68, 1.10)	0.61 (0.46, 0.80)	1.02 (0.70, 1.51)	
Yes	132	Ref	0.65 (0.31, 1.35)	0.64 (0.28, 1.45)	0.44 (0.12, 1.54)	0.61 (0.17, 2.20)	

Adjusted for gender, age, race, education level, ratio of family income to poverty rate, smoking, hypercholesterolemia, diabetes, cardiovascular disease, stroke, lung disease, failing kidneys, except the subgroup variable. HR, hazard ratio; CI, confidence interval; P inter, P interaction; Ref, reference.

### Relationship between banana intake and all-cause mortality

**Figure 4:**
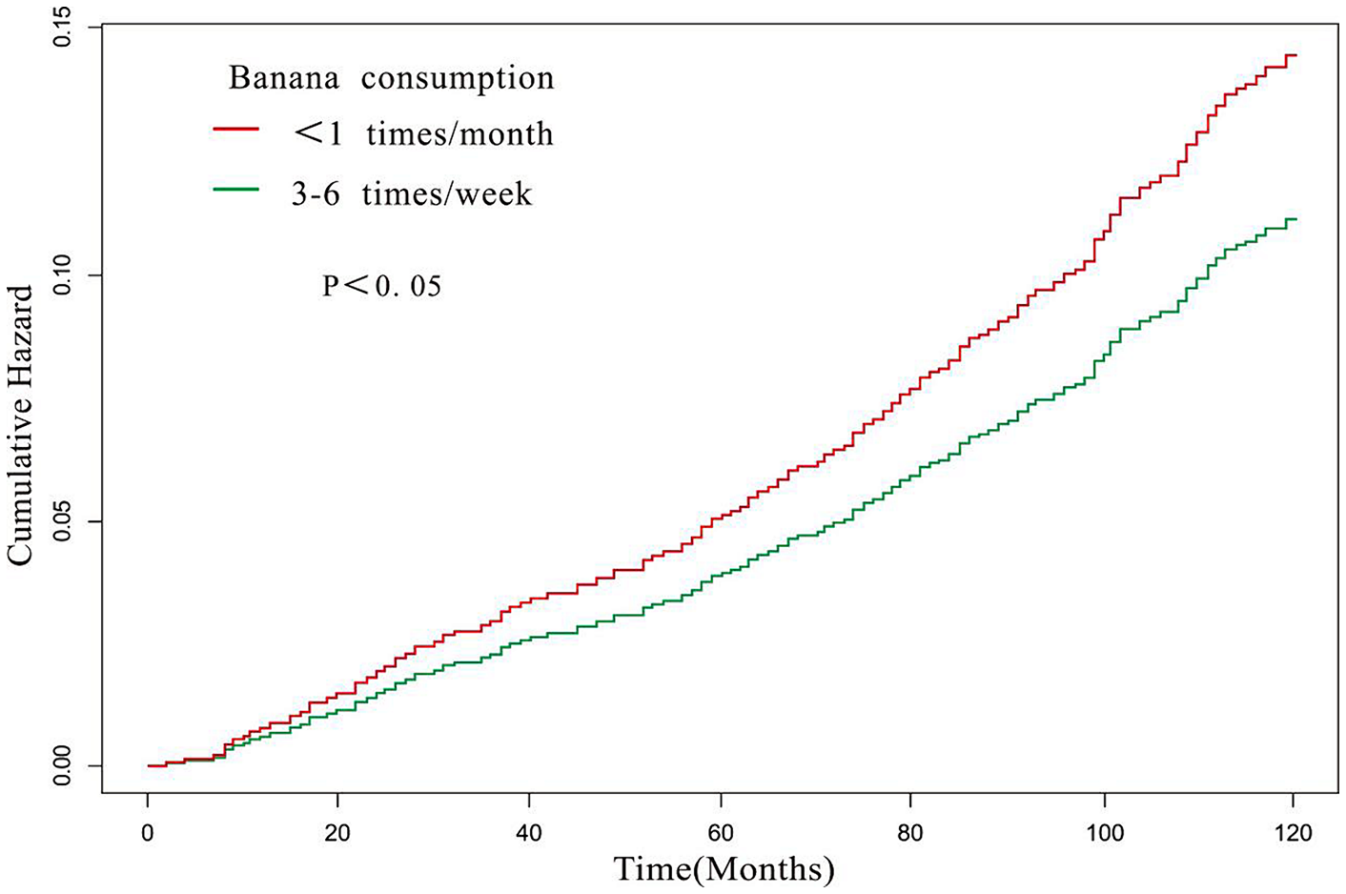
Time-mortality curve of the relationship between banana consumption and all-cause mortality. Adjusted for gender, age, race, education level, ratio of family income to poverty rate, smoking, hypercholesterolemia, diabetes, cardiovascular disease, stroke, lung disease, failing kidneys.

In a subgroup analysis, it was observed that the association between banana consumption and all-cause mortality among individuals with hypertension remained unaffected by the presence or absence of comorbidities (all *p* values for the interaction were > 0.05) (refer to Table 4). By employing COX regression analyses and accounting for confounding factors, the risk ratio for all-cause mortality was calculated. The results demonstrated that hypertensive individuals who consumed bananas three to six times per week had a significantly lower risk of mortality compared to those who consumed bananas less than once per month. The results are shown in Figure 4 (log-rank test *p* < 0.05).

**Table 4:** Stratified analysis of relationship between banana intake and all-cause mortality.

		HR (95%CI)					P inter
	Number	<1 times/month	1–3 times/month	1–2 times/week	3–6 times/week	≥1 times/day	
**Hypercholesterolemia**							0.919
No	1,273	Ref	0.72 (0.48, 1.07)	0.86 (0.59, 1.26)	0.76 (0.53, 1.08)	0.99 (0.67, 1.46)	
Yes	1,207	Ref	0.86 (0.60, 1.24)	0.80 (0.55, 1.15)	0.77 (0.55, 1.09)	1.06 (0.73, 1.53)	
**Diabetes**							0.925
No	1968	Ref	0.76 (0.56, 1.03)	0.81 (0.60, 1.09)	0.75 (0.56, 1.00)	0.96 (0.70, 1.31)	
Yes	512	Ref	0.96 (0.56, 1.66)	0.85 (0.49, 1.48)	0.78 (0.48, 1.25)	1.15 (0.69, 1.92)	
**Cardiovascular Disease**							0.383
No	1972	Ref	0.88 (0.63, 1.25)	0.92 (0.66, 1.28)	0.90 (0.66, 1.23)	1.12 (0.79, 1.57)	
Yes	508	Ref	0.68 (0.44, 1.05)	0.70 (0.46, 1.08)	0.54 (0.36, 0.82)	0.84 (0.55, 1.28)	
**Stroke**							0.404
No	2,264	Ref	0.74 (0.55, 1.00)	0.82 (0.62, 1.10)	0.79 (0.61, 1.04)	0.94 (0.70, 1.26)	
Yes	216	Ref	1.07 (0.57, 1.99)	0.85 (0.45, 1.60)	0.60 (0.31, 1.14)	1.23 (0.66, 2.32)	
**Lung Disease**							0.959
No	1969	Ref	0.82 (0.60, 1.12)	0.86 (0.64, 1.17)	0.76 (0.57, 1.01)	1.04 (0.77, 1.41)	
Yes	511	Ref	0.75 (0.44, 1.26)	0.72 (0.43, 1.20)	0.75 (0.47, 1.19)	0.89 (0.51, 1.52)	
**Liver Condition**							0.117
No	2,380	Ref	0.79 (0.61, 1.04)	0.79 (0.61, 1.03)	0.74 (0.58, 0.95)	0.96 (0.73, 1.26)	
Yes	100	Ref	1.31 (0.20, 8.49)	0.93 (0.14, 5.93)	2.39 (0.41, 14.00)	9.45 (0.96, 93.18)	
**Failing Kidneys**							0.418
No	2,348	Ref	0.73 (0.55, 0.97)	0.76 (0.58, 1.01)	0.71 (0.55, 0.92)	0.98 (0.74, 1.29)	
Yes	132	Ref	1.80 (0.74, 4.36)	1.22 (0.52, 2.87)	1.07 (0.43, 2.66)	1.84 (0.72, 4.65)	

Adjusted for gender, age, race, education level, ratio of family income to poverty rate, smoking, hypercholesterolemia, diabetes, cardiovascular disease, stroke, lung disease, failing kidneys, except the subgroup variable. HR, hazard ratio; CI, confidence interval; P inter, P interaction; Ref, reference.

### Combined consumption of apple and banana with all-cause mortality

**Figure 5:**
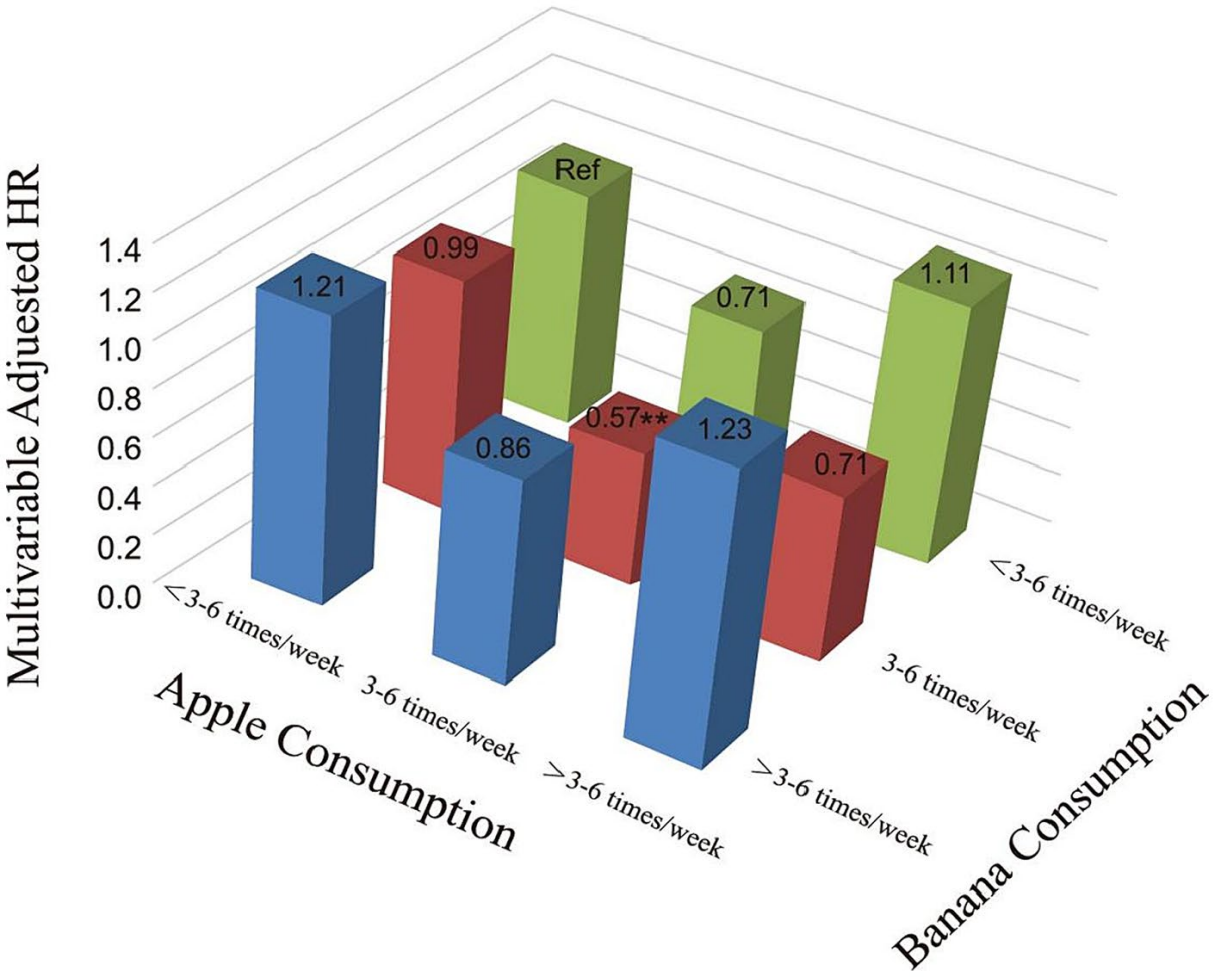
Three-dimensional histogram of relationship of combined consumption of apple and banana with the risk of all-cause mortality. Adjusted for gender, age, race, education level, ratio of family income to poverty rate, smoking, hypercholesterolemia, diabetes, cardiovascular disease, stroke, lung disease, failing kidneys, except the subgroup variable. HR, hazard ratio; Ref, reference. **, *p* < 0.01.

Further research was conducted to investigate the relationship between the consumption of a combination of apples and bananas and all-cause mortality rates. Participants were categorized into nine groups based on their frequency of consuming apples and bananas. Weekly consumption of apples and bananas was recorded as either three to six times, greater than three to six times, or less than three to six times. The Cox proportional hazard model was used to assess the risk of all-cause mortality. The findings (Figure 5; Supplementary Table S2) suggest that consuming apples and bananas three to six times per week significantly reduces the risk of all-cause mortality compared to consuming them less than three to six times per week (HR = 0.57, 95%CI: 0.39–0.84, *p* = 0.005). However, other consumption frequencies did not show a significant reduction in the risk of all-cause mortality.

## Discussion

The study findings demonstrate that consuming apples or bananas three to six times per week is correlated with reduced all-cause mortality among individuals with hypertension, whereas no significant correlation was observed between the consumption of pears, pineapples, and grapes and lower all-cause mortality risks. Additionally, consuming a combination of apples and bananas three to six times per week may be associated with the greatest reduction in the risk of all-cause mortality. According to our knowledge, this is the first study to investigate the association between fruit consumption and all-cause mortality among individuals with hypertension.

Prior studies have demonstrated a connection between the intake of fruits and a decrease in mortality rates among populations afflicted with particular ailments. Chen et al. (8) sought to examine the association between fruit consumption and the dose–response relationship in relation to all-cause mortality in individuals diagnosed with type 2 diabetes in their study. The findings unveiled that a daily fruit consumption exceeding 42.9 g was associated with a 24% reduction in the risk of all-cause mortality (HR = 0.76; 95% CI 0.64–0.88) when compared to those who did not consume fruits. In a retrospective cohort study conducted by Martina et al. (9), it was observed that prostate cancer patients who consumed above-median quantities of fruits and vegetables exhibited a significantly higher likelihood of overall survival at 15 years in comparison to those with lower intake (71% versus 58%, *p* = 0.04; HR = 0.66, 95% CI: 0.47–0.93). These findings indicate a potential association between fruit and vegetable consumption and long-term survival among individuals diagnosed with prostate cancer. Similar to the aforementioned conditions, hypertension is a noteworthy health issue that affects a considerable portion of the population. Numerous studies have provided evidence that the consumption of fruit can reduce blood pressure levels in individuals with hypertension. However, the potential influence of fruit consumption on mortality rates among hypertensive populations has yet to be established. Our findings indicate that a moderate intake of apples and bananas is associated with a reduction in all-cause mortality among patients with hypertension, implying that fruit consumption may also confer survival benefits to hypertensive individuals.

Numerous studies have elucidated the positive impact of fruit consumption on health (23, 24). A meta-analysis has revealed that increased consumption of vegetables and fruits is associated with reduced all-cause mortality rates (24). The consumption of five servings of vegetables and fruits per day reached a point at which all-cause mortality no longer decreased. Our research suggests that consuming apples and bananas three to six times per week is associated with a decrease in mortality rates. However, increasing consumption beyond this range did not provide additional advantages. Further investigation into the relationship between fruit consumption and health is necessary to develop updated guidelines for optimal fruit intake.

Previous studies have primarily focused on the association between overall fruit consumption and mortality rates, with limited examination of the effects of specific types of fruit. Jonathan et al. found that increased apple consumption was associated with reduced all-cause mortality in elderly women (25). Our own research further supports this notion, demonstrating that the proper consumption of apples can effectively mitigate the risk of mortality. Jonathan’s study demonstrates that there is no significant correlation between heightened banana consumption and all-cause mortality. Conversely, our study has identified a notable association between appropriate intake of bananas and all-cause mortality. Discrepancies in findings may be ascribed to multiple factors observed across various studies, including disparities in the study population, methods of measuring fruit consumption, adjustments for confounding variables, duration of follow-up, and characteristics of the cohort.

There is a lack of literature describing individual fruits, which makes it difficult to understand the mechanisms involved. Both apples and pears belong to the Rosaceae family and are excellent sources of dietary fiber and phenolic compounds (26, 27). Dietary fiber has been shown to have a positive impact on cholesterol levels, blood sugar, and blood pressure, as well as promoting a healthy intestinal flora (28, 29). Phenolic compounds possess antioxidant, anti-inflammatory, anti-cancer, anti-aging, antiviral (30, 31). The study found no significant difference in dietary fiber content between apples and pears. However, apple skin had the highest concentration of polyphenolic compounds. In terms of inhibiting DPPH free radicals, the apple peel extract was the strongest while the pear pulp extract was the weakest (32). A study found that apples are the primary source of phenolic compounds and antioxidants in Northern Europe and the United States (33). The potential health benefits of apple polyphenols may exceed those of pear polyphenols. The antioxidant effects of pear polyphenols have only been demonstrated *in vitro*, as they have received less attention in research (26). In addition to this, apples can alleviate the symptoms of chronic diseases and reduce the risk of cardiovascular disease and cancer, due in large part to apple polyphenols (27, 34). As one of the most commonly consumed fruits, grapes are rich in antioxidants such as polyphenols and flavonoids. These antioxidants can reduce atherosclerosis by inhibiting the oxidation of low-density lipoproteins and activating novel proteins that prevent cellular senescence. Additionally, they can help prevent cardiovascular disease (35, 36). However, compared to apples, grapes may contain less dietary fiber. Dietary fiber has a blood pressure-lowering effect, which may be one of the reasons why apples can help alleviate chronic diseases (28, 29). Pineapple contains polyphenols, dietary fiber, and minerals. Studies have shown that pineapple exhibits anti-inflammatory and antioxidant activity (37). The polyphenol content in pineapple is lower than in apples. Although some studies have shown that pineapple skin contains more bioactives, it is not edible. Therefore, it is recommended to consume processed pineapple products and extracts (38). As a highly popular fruit worldwide, bananas possess a diverse range of nutrients, including phenolic compounds, vitamins C and E, carotenoids, as well as potassium ions (39). Many of these compounds exhibit antioxidant activity that protects the body against oxidative stresses, potentially decreasing mortality rates (40). Vitamin E, vitamin C, and carotenoids react with free radicals, specifically peroxyl radicals, and monolinear molecular oxygen to achieve antioxidant effects (19). Vitamin E helps prevent the oxidation of unsaturated fatty acids to peroxides, while carotenoids possess the ability to scavenge single-linear oxygen. Vitamin C plays a role in the regeneration of vitamin E and the elimination of free radicals within the cytoplasm (19). Furthermore, vitamin E has the potential to decrease platelet aggregation by inhibiting the proliferation of smooth muscle cells and reducing platelet adhesion to collagen (41). When comparing the potassium content of commonly consumed fruits, bananas contain more potassium than pears, and green grapes (17). Studies have shown that the consumption of potassium from dietary sources can lead to a decrease in blood pressure and a reduction in overall mortality (42–45). These mechanisms may help explain the correlation between bananas and apples intake and all-cause mortality, but not other fruits, with lowered all-cause mortality in our results for hypertensive patients. However, it is important to note that the conclusions of this study are specific to a particular population and cohort. Therefore, they should not be used to dismiss the health benefits of other fruits.

### Strengths and weaknesses

An important strength of this study is that it examines the association between fruit consumption and all-cause mortality within a hypertensive population, thereby providing a unique contribution to existing literature that has primarily focused on the general population. Moreover, our study boasts a substantial and diverse sample size, encompassing participants from different regions of the United States, varying ethnic backgrounds, and diverse socioeconomic statuses. The selection of the NHANHS database was based on its reputation for providing high-quality data and its recognition within the field. However, the scope of our study is constrained as it solely focused on examining the effects of apple and banana consumption on all-cause mortality, without considering the associations with specific causes of mortality such as cardiovascular disease, cancer, or chronic conditions. The researchers assessed the fruit intake of participants, which may introduce random measurement errors due to variations in individual diets over time. Additionally, observational studies may be subject to bias. In our study, we adjusted for covariates such as education level, poverty rate, and smoking to account for potential confounding factors. Although we made diligent attempts to account for all potential confounding variables and mitigate the impact of covariates in our analysis, it is plausible that unidentified or unmeasured factors could have influenced our findings.

## Conclusion

In summary, our study has revealed that the consumption of a suitable quantity of apples or bananas can effectively lower mortality rates among individuals diagnosed with hypertension. Furthermore, the synergistic effect of consuming both fruits may yield even more advantageous outcomes. These findings may contribute novel evidence to the existing body of knowledge on dietetics for hypertensive populations.

## Data availability statement

The original contributions presented in the study are included in the article/Supplementary material, further inquiries can be directed to the corresponding authors.

## Ethics statement

The studies involving humans were approved by the Ethics Review Board of the National Center for Health Statistics. The studies were conducted in accordance with the local legislation and institutional requirements. The participants provided their written informed consent to participate in this study.

## Author contributions

CS: Data curation, Formal analysis, Methodology, Writing – original draft. JL: Data curation, Formal analysis, Methodology, Writing – original draft. ZZ: Methodology, Writing – original draft. SR: Validation, Writing – original draft. YG: Writing – original draft. MZ: Validation, Writing – original draft. TL: Validation, Writing – original draft. LT: Validation, Writing – original draft. QY: Project administration, Supervision, Writing – review & editing. LC: Conceptualization, Project administration, Supervision, Writing – review & editing.

## Footnotes

^1^ http://www.Empowerstats.com

## References

[1] Di Renzo L Gualtieri P De Lorenzo A. Diet, nutrition and chronic degenerative diseases. Nutrients. (2021) 13:1372. doi: 10.3390/nu13041372, PMID: 33923865 PMC8072879

[2] Wallace TC Bailey RL Blumberg JB Burton-Freeman B Chen CO Crowe-White KM. Fruits, vegetables, and health: a comprehensive narrative, umbrella review of the science and recommendations for enhanced public policy to improve intake. Crit Rev Food Sci Nutr. (2020) 60:2174–211. doi: 10.1080/10408398.2019.1632258, PMID: 31267783

[3] Zhan J Liu YJ Cai LB Xu FR Xie T He QQ. Fruit and vegetable consumption and risk of cardiovascular disease: a meta-analysis of prospective cohort studies. Crit Rev Food Sci Nutr. (2017) 57:1650–63. doi: 10.1080/10408398.2015.1008980, PMID: 26114864

[4] Hebden L O'Leary F Rangan A Singgih Lie E Hirani V Allman-Farinelli M. Fruit consumption and adiposity status in adults: a systematic review of current evidence. Crit Rev Food Sci Nutr. (2017) 57:2526–40. doi: 10.1080/10408398.2015.1012290, PMID: 26115001

[5] Du H Li L Bennett D Guo Y Turnbull I Yang L. Fresh fruit consumption in relation to incident diabetes and diabetic vascular complications: a 7-y prospective study of 0.5 million Chinese adults. PLoS Med. (2017) 14:e1002279. doi: 10.1371/journal.pmed.1002279, PMID: 28399126 PMC5388466

[6] Aune D Giovannucci E Boffetta P Fadnes LT Keum N Norat T. Fruit and vegetable intake and the risk of cardiovascular disease, total cancer and all-cause mortality-a systematic review and dose-response meta-analysis of prospective studies. Int J Epidemiol. (2017) 46:1029–56. doi: 10.1093/ije/dyw319, PMID: 28338764 PMC5837313

[7] Hu D Huang J Wang Y Zhang D Qu Y. Fruits and vegetables consumption and risk of stroke: a meta-analysis of prospective cohort studies. Stroke. (2014) 45:1613–9. doi: 10.1161/STROKEAHA.114.004836 24811336

[8] Chen Y Su J Qin Y Luo P Shen C Pan E. Fresh fruit consumption, physical activity, and five-year risk of mortality among patients with type 2 diabetes: a prospective follow-up study. Nutr Metab Cardiovasc Dis. (2022) 32:878–88. doi: 10.1016/j.numecd.2021.10.024, PMID: 35078677

[9] Taborelli M Polesel J Parpinel M Stocco C Birri S Serraino D. Fruit and vegetables consumption is directly associated to survival after prostate cancer. Mol Nutr Food Res. (2017) 61:1600816. doi: 10.1002/mnfr.201600816, PMID: 27805317

[10] Al-Makki A DiPette D Whelton PK Murad MH Mustafa RA Acharya S. Hypertension pharmacological treatment in adults: a World Health Organization guideline executive summary. Hypertension. (2022) 79:293–301. doi: 10.1161/HYPERTENSIONAHA.121.18192, PMID: 34775787 PMC8654104

[11] Charchar FJ Prestes PR Mills C Ching SM Neupane D Marques FZ. Lifestyle management of hypertension: International Society of Hypertension position paper endorsed by the world hypertension league and European Society of Hypertension. J Hypertens. (2024) 42:23–49. doi: 10.1097/HJH.0000000000003563, PMID: 37712135 PMC10713007

[12] Filippou CD Tsioufis CP Thomopoulos CG Mihas CC Dimitriadis KS Sotiropoulou LI. Dietary approaches to stop hypertension (DASH) diet and blood pressure reduction in adults with and without hypertension: a systematic review and Meta-analysis of randomized controlled trials. Adv Nutr. (2020) 11:1150–60. doi: 10.1093/advances/nmaa041, PMID: 32330233 PMC7490167

[13] Juraschek SP Miller ER 3rd Weaver CM Appel LJ. Effects of sodium reduction and the DASH diet in relation to baseline blood pressure. J Am Coll Cardiol. (2017) 70:2841–8. doi: 10.1016/j.jacc.2017.10.011, PMID: 29141784 PMC5742671

[14] Filippou CD Thomopoulos CG Kouremeti MM Sotiropoulou LI Nihoyannopoulos PI Tousoulis DM. Mediterranean diet and blood pressure reduction in adults with and without hypertension: a systematic review and meta-analysis of randomized controlled trials. Clin Nutr. (2021) 40:3191–200. doi: 10.1016/j.clnu.2021.01.030, PMID: 33581952

[15] Guasch-Ferré M Willett WC. The Mediterranean diet and health: a comprehensive overview. J Intern Med. (2021) 290:549–66. doi: 10.1111/joim.13333, PMID: 34423871

[16] Slomski A. Mediterranean diet vs low-fat diet for patients with heart disease. JAMA. (2022) 327:2386. doi: 10.1001/jama.2022.9509, PMID: 35762998

[17] Slavin JL Lloyd B. Health benefits of fruits and vegetables. Adv Nutr. (2012) 3:506–16. doi: 10.3945/an.112.002154, PMID: 22797986 PMC3649719

[18] Li B Li F Wang L Zhang D. Fruit and vegetables consumption and risk of hypertension: a Meta-analysis. J Clin Hypertens (Greenwich). (2016) 18:468–76. doi: 10.1111/jch.12777, PMID: 26826021 PMC8032179

[19] Sies H Stahl W Sundquist AR. Antioxidant functions of vitamins. Vitamins E and C, beta-carotene, and other carotenoids. Ann N Y Acad Sci. (1992) 669:7–20. doi: 10.1111/j.1749-6632.1992.tb17085.x 1444060

[20] Margetts B. FAO/WHO launch expert report on diet, nutrition and prevention of chronic diseases. Public Health Nutr. (2003) 6:323–5. doi: 10.1079/PHN2003481, PMID: 12795818

[21] McGuire S. Scientific report of the 2015 dietary guidelines advisory committee. Washington, DC: US Departments of agriculture and health and human services, 2015. Adv Nutr. (2016) 7:202–4. doi: 10.3945/an.115.011684, PMID: 26773024 PMC4717899

[22] Yang YX Wang XL Leong PM Zhang HM Yang XG Kong LZ. New Chinese dietary guidelines: healthy eating patterns and food-based dietary recommendations. Asia Pac J Clin Nutr. (2018) 27:908–13. doi: 10.6133/apjcn.072018.03, PMID: 30045438

[23] Miller V Mente A Dehghan M Rangarajan S Zhang X Swaminathan S. Fruit, vegetable, and legume intake, and cardiovascular disease and deaths in 18 countries (PURE): a prospective cohort study. Lancet. (2017) 390:2037–49. doi: 10.1016/S0140-6736(17)32253-5 28864331

[24] Wang X Ouyang Y Liu J Zhu M Zhao G Bao W. Fruit and vegetable consumption and mortality from all causes, cardiovascular disease, and cancer: systematic review and dose-response meta-analysis of prospective cohort studies. BMJ. (2014) 349:g4490. doi: 10.1136/bmj.g4490, PMID: 25073782 PMC4115152

[25] Hodgson JM Prince RL Woodman RJ Bondonno CP Ivey KL Bondonno N. Apple intake is inversely associated with all-cause and disease-specific mortality in elderly women. Br J Nutr. (2016) 115:860–7. doi: 10.1017/S0007114515005231, PMID: 26787402

[26] Reiland H Slavin J. Systematic review of pears and health. Nutr Today. (2015) 50:301–5. doi: 10.1097/NT.0000000000000112, PMID: 26663955 PMC4657810

[27] Zhang Y Zeng M Zhang X Yu Q Zeng W Yu B. Does an apple a day keep away diseases? Evidence and mechanism of action. Food Sci Nutr. (2023) 11:4926–47. doi: 10.1002/fsn3.3487, PMID: 37701204 PMC10494637

[28] Veronese N Solmi M Caruso MG Giannelli G Osella AR Evangelou E. Dietary fiber and health outcomes: an umbrella review of systematic reviews and meta-analyses. Am J Clin Nutr. (2018) 107:436–44. doi: 10.1093/ajcn/nqx082, PMID: 29566200

[29] Reynolds AN Akerman A Kumar S Diep Pham HT Coffey S Mann J. Dietary fibre in hypertension and cardiovascular disease management: systematic review and meta-analyses. BMC Med. (2022) 20:139. doi: 10.1186/s12916-022-02328-x 35449060 PMC9027105

[30] Stevenson DE Hurst RD. Polyphenolic phytochemicals – just antioxidants or much more? Cell Mol Life Sci. (2007) 64:2900–16. doi: 10.1007/s00018-007-7237-1 17726576 PMC11136160

[31] Rahman MM Rahaman MS Islam MR Rahman F Mithi FM Alqahtani T. Role of phenolic compounds in human disease: current knowledge and future prospects. Molecules. (2021) 27:233. doi: 10.3390/molecules27010233 35011465 PMC8746501

[32] Leontowicz M Gorinstein S Leontowicz H Krzeminski R Lojek A Katrich E. Apple and pear peel and pulp and their influence on plasma lipids and antioxidant potentials in rats fed cholesterol-containing diets. J Agric Food Chem. (2003) 51:5780–5. doi: 10.1021/jf030137j, PMID: 12952433

[33] Commisso M Bianconi M Poletti S Negri S Munari F Ceoldo S. Metabolomic profiling and antioxidant activity of fruits representing diverse apple and pear cultivars. Biology (Basel). (2021) 10:380. doi: 10.3390/biology10050380 33924913 PMC8145694

[34] Knekt P Kumpulainen J Järvinen R Rissanen H Heliövaara M Reunanen A. Flavonoid intake and risk of chronic diseases. Am J Clin Nutr. (2002) 76:560–8. doi: 10.1093/ajcn/76.3.560 12198000

[35] Dohadwala MM Vita JA. Grapes and cardiovascular disease. J Nutr. (2009) 139:1788s–93s. doi: 10.3945/jn.109.107474, PMID: 19625699 PMC2728695

[36] Ruf JC. Wine and polyphenols related to platelet aggregation and atherothrombosis. Drugs Exp Clin Res. (1999) 25:125–31. PMID: 10370875

[37] Mohd Ali M Hashim N Abd Aziz S Lasekan O. Pineapple (*Ananas comosus*): a comprehensive review of nutritional values, volatile compounds, health benefits, and potential food products. Food Res Int. (2020) 137:109675. doi: 10.1016/j.foodres.2020.109675 33233252

[38] Zhao Q Ge Q Shang Y Zheng M Sun X Bao S. Eating with peel or not: investigation of the peel consumption situation and its nutrition, risk analysis, and dietary advice in China. Food Res Int. (2023) 170:112972. doi: 10.1016/j.foodres.2023.112972, PMID: 37316012

[39] Singh B Singh JP Kaur A Singh N. Bioactive compounds in banana and their associated health benefits – a review. Food Chem. (2016) 206:1–11. doi: 10.1016/j.foodchem.2016.03.033, PMID: 27041291

[40] Aune D Keum N Giovannucci E Fadnes LT Boffetta P Greenwood DC. Dietary intake and blood concentrations of antioxidants and the risk of cardiovascular disease, total cancer, and all-cause mortality: a systematic review and dose-response meta-analysis of prospective studies. Am J Clin Nutr. (2018) 108:1069–91. doi: 10.1093/ajcn/nqy097, PMID: 30475962 PMC6250988

[41] Eichholzer M Lüthy J Gutzwiller F Stähelin HB. The role of folate, antioxidant vitamins and other constituents in fruit and vegetables in the prevention of cardiovascular disease: the epidemiological evidence. Int J Vitam Nutr Res. (2001) 71:5–17. doi: 10.1024/0300-9831.71.1.5, PMID: 11276922

[42] Whelton PK He J Cutler JA Brancati FL Appel LJ Follmann D. Effects of oral potassium on blood pressure. Meta-analysis of randomized controlled clinical trials. JAMA. (1997) 277:1624–32. doi: 10.1001/jama.1997.03540440058033 9168293

[43] Dickinson HO Nicolson DJ Campbell F Cook JV Beyer FR Ford GA. Magnesium supplementation for the management of essential hypertension in adults. Cochrane Database Syst Rev. (2006) 3:Cd004640. doi: 10.1002/14651858.CD004640.pub2, PMID: 16856052 PMC13429907

[44] Kwon YJ Lee HS Park G Lee JW. Association between dietary sodium, potassium, and the sodium-to-potassium ratio and mortality: a 10-year analysis. Front Nutr. (2022) 9:1053585. doi: 10.3389/fnut.2022.1053585, PMID: 36438773 PMC9691953

[45] Bagheri A Naghshi S Sadeghi O Larijani B Esmaillzadeh A. Total, dietary, and supplemental magnesium intakes and risk of all-cause, cardiovascular, and Cancer mortality: a systematic review and dose-response Meta-analysis of prospective cohort studies. Adv Nutr. (2021) 12:1196–210. doi: 10.1093/advances/nmab001, PMID: 33684200 PMC8321838

