# Supplementary material for: The correlation between fruit intake and all-cause mortality in hypertensive patients: a 10-year follow-up study
Source: Front Nutr. 2024 Mar 22;11:1363574. doi: 10.3389/fnut.2024.1363574 (PMC10995410; doi:10.3389/fnut.2024.1363574)
Supplement: Supplementary file 1 [file Table_1.DOCX]

Table S1. Website for database acquisition

| Data | The official website |
| --- | --- |
| NHANES | https://www.cdc.gov/nchs/nhanes/ |
| NDI | https://www.cdc.gov/nchs/data-linkage/mortality.htm |

Table S2. Hazard ratios and confidence intervals of all-cause mortality for apple and banana consumption

| **Banana**  **Apple** | **HR（95%CI）P** | | |
| --- | --- | --- | --- |
|  | ＜3-6 times/week | 3-6 times/week | ＞3-6 times/week |
| ＜3-6 times/week | Ref. | 0.99 (0.79, 1.23) 0.914 | 1.21 (0.94, 1.56) 0.147 |
| 3-6 times/week | 0.71 (0.45, 1.10) 0.128 | 0.57 (0.39, 0.84) 0.005 | 0.86 (0.53, 1.41) 0.560 |
| ＞3-6 times/week | 1.11 (0.56, 2.18) 0.764 | 0.71 (0.35, 1.45) 0.348 | 1.23 (0.72, 2.09) 0.449 |

Abbreviations: HR, hazard ratio; CI, confidence interval; P inter, P interaction; Ref., Reference.

******:*P*<0.01

Adjusted for gender, age, race, education level, ratio of family income to poverty rate, smoking, hypercholesterolemia, diabetes, cardiovascular disease, stroke, lung disease, failing kidneys
